# Islam and Mental Disorders of the Older Adults: Religious Text, Belief System and Caregiving Practices

**DOI:** 10.1007/s10943-020-01094-5

**Published:** 2020-11-03

**Authors:** Suhad Daher-Nashif, Suzanne H. Hammad, Tanya Kane, Noor Al-Wattary

**Affiliations:** 1 The College of Medicine, QU Health, Qatar University, Doha, Qatar; 2 Centre for Humanities and Social Sciences, Qatar University, Doha, Qatar

**Keywords:** Islam, Older adults, Dementia, Caregiving, Middle East

## Abstract

This paper illustrates the impact of Islamic religious texts on dementia care in the Middle East. It examines how old age and older adults mental disorders are framed in the Quran and Hadith, and how these texts are transformed to belief ideologies and caregiving practices. The study uses a qualitative research methods, which include a review of all Islamic holy texts that address mental and cognitive changes associated with ageing, along with interviews with eight Sharia scholars and 37-Arab-Muslim families living in Qatar. Islamic texts command compassion and honouring of elderly parents and give care instructions. These texts are transformed into social practices and used as diagnostic and treatment tools.

## Introduction

Religion can have an important influence on human health and behaviour, and people draw on religious resources when coping with illness and life stressors (Miller and Thoresen 2003). Religious and spiritual views also affect how individuals from certain cultural groups view neurocognitive disorders, such as dementia-related symptoms (Sayegh and Knight 2013).^1^ Some of these beliefs can lead to a delay in seeking help or to not seeking help from formal healthcare systems (Dilworth-Anderson and Gibson 2002; Sayegh and Knight 2013). Several researchers^2^ have highlighted the importance of an awareness of cultural and religious perceptions to assess the unique health needs of individuals. The Health Belief Model (Rosenstock et al. 1988), for example, considers religion and beliefs as significant variables affecting perceptions of illness and health, with a major impact on managing health and sickness in certain societies. When a researcher discusses religious impact on human behaviours, he/she must take in consideration the three layers of religion: the holy text,^3^ the belief system derived from the text, and the social-religious practices derived from both and influenced by the local culture (Abu-Lughod 1993 [1997]). Furthermore, in several contexts, there may be gaps between the holy text, the belief system and the practice. Written texts have a social role and agency to structure the social lives of human beings (Latour 2005) and have an active contribution to the discourse surrounding social issues (Cooren 2004). The focus of this study is on how Islamic religious texts contribute to religious discourse and social behaviour surrounding ageing and mental disorders of the older people in Arab-Muslim society, with particular reference to dementia care in the Middle East.

### Ageing and Mental Illness in Arab-Muslim Societies

Neurocognitive skills and abilities are recognised in Islam (Tumiran et al. 2018). Examples include the ability to distinguish between good and bad at the age of seven under normal cognitive developmental processes and the ability to think sanely, called *a’ql.*^4^ These cognitive capacities are pre-conditions for acceptable prayers (Tumiran et al. 2018). In Arab-Muslim societies, the most common word to describe dementia is *Kharaf*, which means ‘unravelled’ or ‘loss of mind’. It holds a negative connotation and is mostly perceived as an inevitable stage associated with ageing (Cohen et al. 2009). According to the Islamic perspective,^5^ the most distinctive symptom of senile dementia is the loss of memory and subsequent decline in cognitive ability (Al-Razi 1990).^6^ Mental disorders are still perceived by some groups in Arab-Muslim communities as a test or punishment from God (Abu-Ras et al. 2008; Rassool 2000); others view it as a predetermined destiny chosen by God and thus accept it with optimism and a desire for treatment (Nabolsi and Carson 2011). For others, mental illness is perceived as an opportunity to remedy a disconnection from religion or a lack of faith through regular prayer and a sense of personal responsibility (Padela et al. 2012; Youssef and Deane 2006). The studies on dementia in Arab communities pointed to the role of strong support systems for older age persons and the positive influence of extended families’ support and faith in cementing this role (Abdelmoneium and Alharahsheh 2016).

A recent study conducted among the Palestinian minority living in Israel provided new insights into the relationships and emotions of caregivers towards those in their care. The study revealed positive feelings and continuous support towards persons with Alzheimer’s dementia; these emotions include both sympathy and empathy for the older adults’^7^ difficult situation and mercy and compassion (Abojabel and Werner 2016). In Arab-Muslim societies, the older people are often perceived as a source of spiritual blessing, religious faith, wisdom and love (Cipriani and Borin 2015), and respecting them is associated with the principle of filial piety, which is an integral part of Arab culture (Khalaila 2010). As a result, sometimes mental disorders are treated as a ‘private family issue’ and religious alternative tools, such as consultation with a sheikh, are used in the treatment process (Hamdan 2009). Although sheikhs and imams^8^ do not have formal training in addressing mental disorders (Ciftci et al. 2013), they play significant roles in counselling families dealing with mental issues (Abu-Ras et al. 2008; Youssef and Deane 2006).

However, it has been found that in Arab communities there is often a stigma associated with mental illness. For example, Ghuloum et al. (2010) found that fewer than half of their study subjects (Arabs living in Qatar) believed that mentally ill people are “mentally retarded”^9^ (40.6%) and 48.3% believed that mental illness is a punishment from God. The questions that arise here are: What are the religious sources for these attitudes, if any and how do these perceptions affect the caregiving practices for older age persons with a mental disorder, in this case, dementia? These questions and more will be addressed in this study.

## Methodology

Examining meanings, attitudes and socio-religious practices requires a qualitative methodology that explains the link between the different factors affecting human behaviour, in this case, religious texts and the belief system. For this study, a grounded theory approach to qualitative exploratory design was used to develop a better in-depth understanding of this link.

### Data Collection

The qualitative tools used in collecting the data include written texts and semi-structured interviews.

#### Islamic Texts

The study uses an indexed dictionary^10^ of the Quran^11^ to search for verses mentioning the words older adults, sheikh, mental, mental illness, mental disorder, sickness, madness, senile, weakness and parents.^12^ All verses mentioned in previous studies and by Sharia^13^ scholars were also reviewed. In addition to the Quran, the Hadith^14^ was reviewed by searching for the same words in Sahih Al-Bukhari and Sahih Muslim, the most trusted Hadith collections used by Sunni Muslims.

#### Interviews

Semi-structured interviews were conducted with eight Sharia scholars, five of whom were Fiqh^15^ researchers, as well as one imam and two female religious instructors within the community. Furthermore, one mufti’s^16^ presentation on dementia in Islam was observed at a local conference in Qatar. In addition, 37 caregivers were interviewed as part of a project on dementia care in Qatar,^17^ with questions pertaining to religious practices and beliefs surrounding care for old adults with dementia. In order to reach Sharia scholars for the interviews, we asked social science experts in Qatar about local leading figures known for seeking out, teaching and spreading knowledge on Sharia. Recommendations led us to four primary figures, who in turn directed us to more interviewees.

In order to reach families for interviews, we received the help of Ehsan-Centre for Empowerment and Elderly Care in Qatar. Those who affirmed initial acceptance to be interviewed were contacted for formal consent and scheduling of an interview. In addition, a snowball approach was used with families, where some interviewees helped us to reach another family who expressed initial interest in being interviewed. Interviews with male Sharia scholars were conducted at their workplaces, while interviews with female Sharia instructors were conducted at their homes. Interviews with families were conducted at homes. Interviewees decided on the date and time of the interviews. Most of the interviews were conducted in Arabic, the native language of the interviewees. All interviews were transcribed. The main author translated the relevant sections on religion from the original interviews and gave it to a professional translator and editor to translate it back to Arabic. Both parties were involved in discussion until a satisfactory agreement was reached regarding any gaps of information and differences of interpretation.

### Ethical Assurance

Qatar University IRB approved this study. Onsite informed consent was obtained from all participants. Interviews were recorded when given permission by the interviewees, but if an interviewee declined recording, the interviewer documented the interview manually. Interviewees could request stopping the recording at any stage of the interview and were allowed to withdraw at any stage of the study. For confidentiality assurance, the study uses pseudonyms, and no identifiable details were used in documenting the findings.

### Data Analysis

The study followed Strauss and Corbin’s (1998) grounded theory approach for content analysis. This included open, axial and selective coding of the texts and interviews. The main author and two co-authors read the data twice to determine patterns and categories.

The content analyses of the texts and interviews resulted in four main themes of analysis: (a) meaning and characteristics of older adults; (b) framing dementia in older age persons; and (c) caring for older adults with dementia.

## Findings

This section outlines the three primary themes emerged from the content analysis. Each theme first outlines the religious texts from the Quran and Hadith, second the belief system as framed by the Sharia scholars and then the social practices as expressed by the family caregivers. The aim is to highlight the link between the three religions systems, and how one is derived from the other.

### Meaning and Characteristics of Older Adults

The term ‘Sheikh’ means ‘old person’ in Arabic, but in the Quran and Hadith, it has two meanings: advanced in religious knowledge and advanced in age. The first interpretation of the word indicates knowledge and wisdom and is associated with experiences accumulated over a lifetime. The word Sheikh as old age appears in four places in the Quran. One example is:

She^18^ said, ‘Alas for me. Shall I give birth, when I am an old woman, and this, my husband, is an old man? This is truly a strange thing.’ (Quran 11:72)

This verse frames older age persons as those who can no longer procreate; thus, ageing in this verse is linked to physical, not mental, (dis)ability.

Another example is:

It is He who created you from dust, then from a seed, then from an embryo, then He brings you out as an infant, then He lets you reach your maturity, then you become elderly—although some of you die sooner—so that you may reach a predetermined age, so that you may understand. (Quran 40:67)

This verse reflects how developmental stages are described in the Quran, where being an old adult is framed as closeness to death. The description of each developmental stage indicates characteristics and definition of older age in the Quran.

Developmental stages also appear in Surah 46, where the age of strength is given as 40, from which weakness also begins:

And We have enjoined upon man, to his parents, good treatment. His mother carried him with hardship and gave birth to him with hardship, and his gestation and weaning [period] is thirty months. [He grows] until, when he reaches maturity and reaches [the age of] forty years, he says, “My Lord, enable me to be grateful for Your favor which You have bestowed upon me and upon my parents and to work righteousness of which You will approve and make righteous for me my offspring. Indeed, I have repented to You, and indeed, I am of the Muslims.” (Quran 46:15)

Prophet Muhammad (PBUH) framed old age as the only disease that cannot be remedied. This is reflected in the following story: a group of people once asked him, ‘O Messenger of Allah, shall we treat (our ill)?’ He said: ‘Yes, o worshippers of Allah! Use remedies. For indeed Allah did not make a disease but he made a cure for it – or – a remedy, except for one disease’. They said: ‘… What is it?’ He said: ‘Old age’.

The Sharia scholars, who represent the belief system, used most of the above verses and Hadiths when they referred to ageing and mental disorders. They mentioned that being an old adult could be related to age or to (dis)ability. One of the respondents, a researcher in *Fiqh*, said: ‘God Almighty talked about [the] elderly in general after the age of forty’. This fits with Surah 46 verse 15 mentioned above that he also used as reference for his claim. He added:

‘Elderly’ is a relative term. It means that the age of forty doesn’t only signify beginning of weakness, but also wisdom. This is the age the person should consider what will come, because God said: ‘… And We settle in the wombs whatever We will for a designated term, and then We bring you out as infants, until you reach your full strength. And some of you will pass away, and some of you will be returned to the vilest age, so that you may not know, after having known …’. (Quran 22:5)

This Quranic verse refers to the point where regression begins. Sharia scholars agree that ageing and illness in old age are natural developments, unrelated to sins or tests from God. This fits with the verse on development stages mentioned above, in which ageing and regression are natural parts of human development.

### Framing Dementia in Older Age Persons

Characteristics of old age in the Quran include physical and cognitive weaknesses. These are described as follows:

It is Allah who creates you and takes your souls at death; and of you there are some who are sent back to a feeble age, so that they know nothing after having known (much): for Allah is all-Knowing, all-Powerful. (Quran 16:70)

This indicates that cognitive regression is natural part of ageing, and a symbol of God’s power, meaning that not everyone who is a sheikh in age will be a sheikh in knowledge.^19^

In the Quran, senile dementia is mentioned in the Prophet Ya’qub’s (PBUH) story:

When the caravan left (Egypt), their father said: ‘I do indeed smell the presence of Joseph: no, do not think me as a dotard’. They said: ‘By Allah! Truly you are in your old wandering mind’. (Quran 12:94–95)

Ageing and loss of cognitive abilities are mentioned by the Prophet Muhammad (PBUH) as the hardest disease; he even prayed to God to protect him from this disease, saying:

O Allah, I seek refuge with You from cowardice, miserliness, and from being sent back to a feeble age. (Al-Bukhari, Book 16, Hadith 14)

Sharia scholars also confirmed that according to Islam there are signs and characteristics indicating progression in age and regression in mental and/or physical abilities. As described by one Sharia scholar:

Any damage [with]in the human body will affect the psychological status … the weak men, women, and children who have no means to act, God pardon them. … Everything can affect the elderly, even a word, because of that God instructs us to respect them, especially when they reach old age.

He used the verse from Surah 17 as an example.

The Sharia scholars’ perceptions of ageing derive from the religious texts, and transferred to the community in various ways, including books, lectures and Friday sermons in mosques. These perceptions greatly affect the social behaviour of caring for older adults with mental disorder.

Interviews with family caregivers reveal that a number of religious practices and beliefs have penetrated perceptions of dementia and the modes of caring and coping. For many, religious beliefs serve as an explanatory framework for what is happening to their parents, while changes in parents’ practices of religious commandments (such as forgetting time, sequence and content of prayer) can serve as a useful tool to examine change and realise signs of disorder.

The interviews indicated that in the first stages of dementia, families mostly considered mental and cognitive regression to be part of the progression of age. This fits with the religious perception of ageing in the texts and confirms the scholars’ claims above. The interviewees also referred to dementia in three main religious frames: a test from God, a punishment from God that would ease the punishment on doomsday, or a present from God that would guarantee them paradise on the Day of Judgment.

Severe cognitive regression was the sign for families that something wrong is happening with their parent. One third of the caregivers mentioned that they realised that something was seriously wrong when they noticed their parent forgetting prayer times, and especially when they began to forget Quranic verses that they had recited for decades. For example, Sae’d^20^ said:

She began to forget the prayer time when she had never done it before. On the contrary, she was the one to remind us sometimes.

Maha^21^ also said:

In his prayer he added verse and missed some. It was embarrassing because he had no idea, he finished praying and that’s it. He was confused.

Confusion in religious practices functioned as an alert for most families and motivated them to seek formal medical help for their parent.

### Caring for Older Adults with Dementia

The Holy Quran mentions descendants’ duty to care for their parents, and several Quranic verses dictate filial respect. The main verse cited by Sharia scholars in this study was:

The Lord has decreed that you worship none but Him, and that you be kind to parents. Whether one or both of them attain old age in their life, say not to them a word of contempt, nor repel them, but address them in terms of honour. (Quran 17:23)

This verse is followed by another highlighting the special status accorded to parents:

And lower to them the wing of humility, out of mercy, and say, ‘My Lord, have mercy on them, as they raised me when I was a child. (Quran 17:24)

This indicates that respecting parents is a recompense for all they have done for their children.^22^ Prophet Muhammad (PBUH) considered neglecting parents a great sin. As Abu Hurairah narrated^23^:

The Messenger of Allah (PBUH) said: Damn you! Damn you! Damn you! When he was asked, ‘Who was damned, O Messenger of Allah?,’ he replied, ‘He who has elderly parents, or even only one of them is old, but he did not attempt to enter the Heaven by providing good care to his aged parents.’ (Sahih Muslim, 2009)

All Sharia scholars stressed the offspring’s responsibility to care for their parents. The following verse was referenced repeatedly:

The Lord has decreed that you worship none but Him, and that you be kind to parents. Whether one or both of them attain old age in their life, say not to them a word of contempt, nor repel them, but address them in terms of honour.* And lower to them the wing of humility, out of mercy, and say, ‘My Lord, have mercy on them, as they raised me when I was a child.’ (Quran 17:23–24)

One scholar explained that this verse means that after prayer and worship of God the greatest importance lies in treating one’s parents with respect and compassion. The scholars detailed the rules of caregiving as follows: respect, mercy, preference and dignity, friendliness and concern, turning a blind eye to the older adult mistakes and supporting them financially.

The Sharia scholars agreed that religious exemptions for the older adults are based on their physical and mental (dis)abilities, not on their age. They detailed several exemptions for elderly persons with dementia and all mental disorders. One of them said:

In the first stages of Alzheimer’s, when the patient loses the ability to focus, he [she] is still required to perform the commandments. His [her] caregiver should [give] advice and remind the patient; in the second stage, when he [she] becomes forgetful and less aware, the frustrated family has to be patient and understanding; in the third and hardest stage, the patient is totally exempted and the family members can fulfil some of His [God’s] commandments, such as feeding poor people, pilgrimage [on their own or the patient’s behalf], and so on.

Sharia scholars mentioned some religious practices as tools for treatment; praying for the older adults is mentioned as a form of treatment. A female teacher who is also a caregiver for her mother with dementia said:

Saying prayers for your parents is a command from God to the children. … God wants us to treat them with mercy through our prayers. … This is the least we can do for them when they get elderly.

The Sharia scholars highlighted the roles of the family in cases of older age persons with dementia. These roles include reminding them about prayer; greeting, sitting with, talking to and reading verses from the Quran to them; being patient and answering their questions and respectfully listening to their repeated stories; not arguing with them; not sending them to a care home or institution; not leaving the older adult with the nurse or domestic worker all day; and protecting the person with dementia from risks. Furthermore, extended family members have to consider the burden on families. They also mentioned that society has a responsibility towards neglected older adults. The community must intervene so that the older adults do not unduly suffer from illness or loneliness.

Family caregivers reflected their religious duties in caring for their parents. They counted several reasons for caring for their parents with dementia. Strong faith and ‘fear of God’ were two factors mentioned as empowering for the caregivers, some of whom believed that God would reward them for their efforts. Honouring parents and feeling compassion towards them were the most mentioned religious principles in relation to caring for parents with dementia. They asserted their duties using verses 23–24 from Surah 17 mentioned above. Caregivers used the religious commandment *Bir Al walidayn*^24^ to define the motive underpinning care for the older adults, which will be rewarded by God. Additional reward mentioned by caregivers is being cared for by their children when they themselves become older adults. As Fatima^25^ explained:

Those [who are not caring for their parents] will find it in their children’s behaviour towards them. Look at the Prophet (PBUH), he said that, the consequences for most sins we commit may be postponed until the Day of Judgment, except disobedience towards parents; God speeds punishment in one’s lifetime before the Day of Judgment.

Reham^26^ added that she deliberately cares for her mother in front of her three sons and lets them assist, thereby modelling and teaching them. These accounts suggest that the reward promised is not only in the hereafter but also in the present.

Some religious practices are also intervention tools used by the families. More than half of the caregivers mentioned that they read the Quran to the older adults to calm them down. They all believed in the Quran’s power to treat cognitive loss. Samia^27^ said:

When she is nervous and listens to the Quran, she calms down. At night, when she wakes up shouting asking for somebody, I open the Quran and she calms down and falls asleep.

This fits with the religious belief that reading the Quran saves the mind from cognitive regression while also adhering to scholars’ care instructions. For example, Nihal^28^ gave her father the start of a verse, and he could remember and continue to recite the remainder. She said: ‘This was one of the few things that made him happy… seeing him happy also made me happy’. It seems that calming the older adults is a coping mechanism for the caregivers, i.e. reading the Quran for the them is an indirect coping mechanism for the caregivers themselves.

For others, like Haya^29^ and her sisters, the Quran was a form of protection and alternative healing for their mother’s affliction, which they believed was caused by ‘the evil eye’.^30^ Haya said:

My sister went to al-Wakra^31^; she brought an excellent Quran reader because the Quran can protect from evil eye and envy. … She told us that our mother is stricken with evil eye, because she yawned^32^ when she read the Quran.

Religious practices are also used as a coping mechanism to help alleviate the emotional and physical burden of caregiving. In addition to sport and talking to friends, many caregivers said that reading the Quran and praying were the main ways to calm down and relax from the heavy burden of care.

## Discussion

This study has found that there are many similarities between the Islamic texts, the belief system declared by Sharia scholars and the culture of caring for older adults with mental disorder, such as in dementia, within families. This can be explained by the fact that in Muslim communities the religious text constitutes the first reference for understanding life issues, including illness, and serves as a guide to deal with these issues. It is also a reference for information on how God wants people to seek help. Many similarities among the three layers of religion are revealed, especially regarding the meaning of dementia, the processes of ageing and the commandment to care for older adults. However, there are gaps between the caregivers’ perceptions of mental illness as a test or punishment from God and the holy texts and scholars’ framing of it as a natural stage in human development. Mental disorders are still perceived by some groups in Arab-Muslim communities as a test or punishment from God (Rassool 2000; Zolezzi et al. 2017). Sayegh and Knight (2013) noted that these beliefs can cause a delay in diagnosis and seeking medical and institutional help.

Therefore, several researchers (e.g. Cipriani and Borin 2015) suggest conceptualising dementia as a multifactorial process. This approach aligns with Sayegh and Knight (2013), who call for accessible medical care provided by culturally competent professionals to address the values and beliefs that cause people to put off seeking help. They recommend intervention rooted in an evidence-based understanding of a specific social group, rather than health providers’ stereotypes.

The impact of religious beliefs on seeking treatment is in line with the Health Belief Model. Miller and Thoresen (2003) explain how, since the late 1990s, religion and spirituality have been acknowledged as important factors to be included in most research addressing patient and family health issues. In *Religion as a Cultural System*, Clifford Geertz (1966 [2004]) argues that religion has multiple impacts on individuals: it establishes powerful moods and motivations, formulates a general order of existence and makes these conceptions appear as factual and realistic. This study’s findings provide an excellent example of Geertz’s claims, i.e. the Islamic holy texts and belief system offer a legitimated and motivating frameworks for caregivers. Seeing a parent’s illness as a test from God and perceiving caring for him or her as a rewarded behaviour helps caregivers bear the burden, enhances their motivation to care and offers a sense of hope and an unwavering acceptance of God’s decisions. Caring for an afflicted individual is framed in the holy texts as a pathway to paradise; as such, some caregivers perceive dementia as a positive event or as a present from God to guarantee access to heaven. These findings fit with Kane et al. (2020) scoping review on dementia care in the MENA region. Since this study found religious practices to be a tool to examine and a motivating tool for treatment in dementia, healthcare providers working in the MENA region should take note of this approach and incorporate religious practices into diagnostic and intervention processes.

Furthermore, the difficulties experienced by older adults with dementia exempt them from performing religious duties, as mentioned by the Sharia scholars and texts. Tumiran et al. (2018) explain how dementia patients are exempted from the normative duties and responsibilities of adult Muslims, such as performing the five obligatory prayers and fasting during Ramadan.^33^ This is reflected in the social behaviour of caring for the older adults, which indicates the power of the religious text and belief system and their impact on social health and caregiving practices.

## Conclusion

In contexts where nationalities and faiths are diverse, it is crucial to build a system that is attuned to cross-cultural differences. Since the Health Belief Model emphasises family-centred cultural values, healthcare providers’ knowledge of the religious conceptualisation of mental disorders among the older adults, such as dementia, should be a key component of the intervention process. Such familiarity may help minimise cultural resistance to diagnosis and treatment. Furthermore, for social groups where religion is a dominant part of health management, imams based in communities can facilitate the formal health systems in prompting families to seek help. Healthcare providers can benefit from community-based intervention as well as from understanding the religious texts and belief system that shape social health behaviours. This may be a key factor in empowering patient and caregiver’s adherence and alliance to treatment.

## Footnotes

1 For example, it was found that a group of African-Americans attributed dementia to an ‘act of God’ and/or curse that leads to cognitive impairment (Jett 2006). Another example can be found in the research of Zhan (2004), where Chinese-American caregivers linked dementia to punishment for misconduct earlier in life, or ‘bad feng shui’.

2 Such as Cipriani and Borin (2015).

3 Mainly the holy book of each religion.

4 Mind.

5 Most of the published studies discuss the attitudes of Sunni Islam.

6 It is believed that all prophets from Adam to Isa (Jesus) and Muhammad were divinely protected from diseases that affect cognitive abilities, as these might have affected their ability to preach (Tumiran et al. 2018).

7 We follow the WHO terminology and use older adults or older age persons instead elderly, except when the text or interviewee uses the term elderly; then, we do not change it and keep the original term.

8 An imam is the worship leader of a mosque or a Muslim community.

9 The new accepted terminology is intellectual disability.

10 See Abd al-Baqi Muhammad (1994).

11 The holy book for Muslims.

12 In this article, we focus on verses and Hadiths on old-age parents and not on how to behave with parents in general.

13 Islamic law.

14 The statements and actions of the Prophet Muhammad (peace be upon him—henceforth PBUH—an expression used after mentioning a prophet’s name). He is considered as the last prophet by Sunni Muslims. Sunni Islam is the largest denomination of Islam. Its name comes from the word Sunnah, which refers to the Prophet Muhammad’s behaviours and Hadiths.

15 Islamic jurisprudence, or the theory and philosophy of Islamic law.

16 A mufti is a Muslim legal expert who is empowered to give rulings on religious matters and make final decisions on disagreements regarding religious practices.

17 The paper presents part of a study conducted in 2017–2018 on dementia care in Qatar. The study funded by the Centre for Empowerment and Elderly Care-Ehsan and conducted by Qatar University research team (authors of this study). As part of the interview, the interviewees were asked on religious tools and sources for caring for their parents.

18 ‘She’ refers to Sarah, who is known as the Prophet Ishaq’s mother and who became pregnant with Ishaq at the age of 99 after being childless her whole life.

19 Another example of associating ageing with physical weakness appears in Surah 19: He said, ‘My Lord, my bones have become feeble, and my hair is aflame with grey, and never, Lord, have I been disappointed in my prayer to you’. (Quran 19:4) He in this verse refers to the Prophet Zakria who asked God to give him a son when he was 70 years old.

20 A Qatari man, married and caring for his mother with VAD.

21 A daughter of a father and mother with dementia.

22 Another example in Surah 31: ‘We have entrusted the human being with the care of his parents. His mother carried him through hardship upon hardship, weaning him in 2 years. So give thanks to Me, and to your parents. To Me is the destination’ (Quran 31:14).

23 Abu Hurairah was one of those close to the Prophet Muhammad and is considered by Sunni Muslims to be the most prolific narrator of Hadith.

24 Honouring parents.

25 A mother, a religious leader and teacher in her community and daughter of a mother with dementia.

26 A Palestinian-Syrian caregiver for her mother.

27 A single Qatari caregiver.

28 Algerian married to a Qatari man.

29 A single Qatari caregiver.

30 Evil eye is a curse believed to be cast by a malevolent glare, which is usually directed towards a person who is unaware of it. Many cultures believe in the evil eye as a reason for bad luck, illness and injury.

31 Name of a city in Qatar.

32 Some Arab-Muslims believe that if a person yawns when somebody reads them the Quran, this is an indication that they are stricken by the evil eye.

33 The month of fasting.
